# BUILDING A HUMAN REFERENCE ATLAS

**DOI:** 10.1093/geroni/igad104.1556

**Published:** 2023-12-21

**Authors:** Andreas Bueckle

**Affiliations:** Indiana University, Bloomington, Indiana, United States

## Abstract

The Human Reference Atlas (HRA, https://humanatlas.io) is a comprehensive, high-resolution, three-dimensional atlas of all the cells in the healthy human body. The HRA provides standard terminologies and data structures for describing specimens, biological structures, and spatial positions linked to existing ontologies. In this talk, we will present a high-level overview of the major components of the HRA--including 67 anatomically correct 3D Reference Objects for 29 organs and 31 Anatomical Structure, Cell Types, and Biomarker (ASCT+B) Tables--and the tools to explore, use, author, and review the HRA--including the Registration User Interface, the Exploration User Interface, the ASCT+B Reporter, and the HRA Organ Gallery in virtual reality. We welcome experts and practitioners to join the monthly WG meetings (sign up at https://iu.co1.qualtrics.com/jfe/form/SV_bpaBhIr8XfdiNRH), to explore and contribute to this effort, and to provide feedback on the evolving HRA from diverse perspectives.

